# Methadone and neonatal abstinence syndrome (NAS): what we think we know, but do not

**DOI:** 10.3389/fped.2023.1316583

**Published:** 2023-12-21

**Authors:** John J. McCarthy, Loretta P. Finnegan

**Affiliations:** ^1^ Department of Psychiatry, University of California, Davis, CA, United States; ^2^ Finnegan Consulting LLC, Avalon, NJ, United States

**Keywords:** neonatal abstinence syndrome, opioid use disorder, pregnancy, split dosing, rooming-in, methadone metabolism

## Abstract

Since the first use of methadone to treat OUD in pregnancy in the 1970s, there has been a long, controversial, and confusing history of studies, regulatory actions, and practice changes that have clouded an accurate perception of methadone's use in pregnancy. This review will trace this history with a focus on the effect of methadone exposure during pregnancy on neonatal abstinence syndrome (NAS). A new laboratory measure, the serum methadone/metabolite ratio (MMR), has provided a tool for documenting the profoundly dynamic nature of perinatal metabolism. Continuous induction of metabolic enzymes during pregnancy requires dose adjustments and dose frequency changes. The concept of “fetal methadone dosing” emphasizes that relative stability of methadone levels in the fetus is an important consideration for methadone dosing in pregnancy. Finally, the effects of the societal “war on drugs” on pediatric management of neonatal withdrawal risks will be discussed, as well as the importance of comprehensive services for mother and child including the “rooming-in” approach of neonatal care which has considerably replaced the older NICU care model of maternal/infant separation.

## Introduction

A century ago, infants with signs of abstinence were given the diagnosis of congenital morphism (1); they were not provided medication, resulting in an extremely high mortality. It was in the1970's that the infants with *in utero* opioid exposure were given the diagnosis of NAS. Desmond and Wilson described the basics of NAS, what effects onset, the various courses of the syndrome and persistent signs (2). Further, it also became clear that NAS was a potentially a serious medical condition in the newborn since it effected feeding with metabolic complications, inability to sleep, manifestations that led to a comprehensive supportive care approach to mother/fetal/infant unit. Infants were monitored closely for intake and weight gain, fed by gavage if needed, had minimal environmental stimuli (light and noise) and decreased handling, and provided supportive neonatal nursing care (3).

Although Methadone was approved for use in adults with OUD as early as 1946, it was not until a few decades later, in the 1960's that methadone began to be used for pregnant women who had OUD (4). At that time maternal treatment with methadone was thought to be the best approach for treatment of pregnant women with OUD since it was associated with increased prenatal care visits, compliance with program treatment requirements, less risk for medical complications, and was also thought to mitigate the signs of NAS. If the signs became severe, treatment with medication was provided, usually an opioid or sedative or both, and the infant was transferred to the NICU for close monitoring. The assessment of NAS severity required a systematic approach and scoring systems began to be developed and were being used as in adults, in infants with withdrawal signs from prenatal exposure to heroin and/or methadone treatment during pregnancy (5–7). The assessment also helped to identify the infants who needed pharmacologic treatment. Since pharmacologic treatment involved close monitoring of infants in intensive care units resulting in the separation of maternal-infant dyad, the prolonged hospitalization and other side effects of severe NAS fostered the assumption that NAS severity was associated with high methadone doses compared to buprenorphine that was thought to have lesser risk of NAS.

But, does buprenorphine actually cause less neonatal abstinence than methadone? Studies that purport to demonstrate such outcomes suffer from significant limitations. These data are frequently based on hospital records associating methadone dose at delivery to NAS severity and length of hospitalization. Usually, nothing is reported about the actual specifics of treatment with methadone, nor are hospital policies for managing NAS reported beyond morphine dose used to treat NAS, length of hospitalization, and methadone dose at delivery. This review re-assesses what is known about methadone and NAS risk.

### Pharmacokinetics and consequences of maternal/fetal methadone mis-dosing

Missing from virtually all studies are measures of actual fetal methadone exposure as measured by maternal trough serum levels, and absence of any mention of how the medication was taken (i.e., single vs. multiple doses). The fetus is not exposed to the maternal dose, as most outcome studies presume. It is only exposed to the maternal serum level which is reduced by significantly increased metabolic activity due to the continuous induction of CYP450 enzymes by pregnancy hormones (8). Trough serum levels provide the most accurate proxy for fetal exposure. Further, levels maintained within an established therapeutic range (150–600 ng/ml) (9) have been validated as safe and effective in a pregnancy population where all patients were on split doses (10).

Since methadone is converted to an inactive metabolite, maternal and fetal levels of exposure to the active medication can be significantly decreased relative to a non-pregnant population. This metabolic induction begins at conception, and patients conceiving on methadone often report experiencing withdrawal before they realize they are pregnant. The evolutionary goal of this metabolic acceleration is to protect the fetus from toxins. The metabolism of methadone, as well as many other medications, is significantly altered as a result. This requires adaptive dosing strategies, and especially divided dosing.

Historically, however, most pregnant patients have been required to take methadone as a single dose, which exposes both mother and fetus to problematic oscillating serum levels and daily episodes of maternal and fetal withdrawal. The effects of this serum cycling on maternal/fetal stability and NAS have been ignored in virtually all outcome studies. Westermeyer et al. showed that rapid metabolizing patients cannot be effectively stabilized on single methadone doses despite dose increases (11). Use of single doses causes over sedation at the peak serum level (4–6 h after the AM dose) which, because of accelerated metabolism, results in a rapid reduction of serum concentration at the mu receptor, causing withdrawal in the evening and night. This process was documented by ultrasound demonstrating fetal hypomotility at the peak and hypermotility at the trough (12). On divided doses, this physiologic abnormality resolved. Janssen et al. demonstrated fetal cardiac rhythm abnormalities on single doses that also improved on split doses (13).

Women who report breakthrough withdrawal on methadone clearly identify fetal hyperactivity as simultaneously present and which they rate as severe (10). There is animal evidence that, in the fetus, withdrawal activates a catecholamine response that the mother may not mount, suggesting that the fetus may be more sensitive to the adverse effects of withdrawal (14). A study by Rothwell et al, using rodents, found that, in opioid-dependent animals, intermittent opioid exposure (stopping or skipping doses) and related intermittent withdrawal have a role in promoting a modification of brain function and behavior called “psychomotor sensitization” (15). This study used acoustic startle reflexes as a proxy for withdrawal-related stress. Startle potentiation occurred predictably during withdrawal periods. The authors conclude that events that occur during the offset of drug action (i.e., acute withdrawal) may have a pervasive role in adverse effects of opioid exposure. Use of single dose methadone mimics the Rothwell study protocol of frequent on/off receptor occupancy. This process may partly explain why many studies find an association between high methadone dose and NAS severity under conditions where all mothers are maintained on single doses. However, pregnant patients requiring unusually high methadone doses, in the 200–400 mg/day range, have been shown to have serum levels in the therapeutic range and to not have increased NAS risk, provided they are given methadone in multiple divided doses (10). Therefore, rather than dose amount, it may be the single dose regimen, to which most pregnant women have been exposed, that “sensitizes” the fetal brain and potentiates the post-delivery withdrawal response called NAS.

There is further evidence indicating that prenatal fetal stress can alter later hypothalamic-pituitary-adrenal function, behavior, and neurotransmission (14, 16, 17). Recurrent prenatal withdrawal is a type of biologic stress that has been associated with a prolonged surge of corticosteroids (18). Withdrawal is only one of a variety of maternal stressors (physiologic and psychologic) that can adversely affect fetal development via epigenetic alterations of fetal gene expression (19). There is reason for concern that babies exposed to intrauterine withdrawal by single doses, or low dosing practices, or forced or planned withdrawals, may have long term outcomes adversely effected by such intrauterine stress.

Not all mothers on methadone are necessarily rapid metabolizers. A small number have poor genetic loading for metabolic enzymes such that pregnancy induction of metabolism may not affect them or may just move them from poor metabolizers to normal metabolizers. They may feel fine on single doses. However, a strong case can still be made that the fetus needs methadone in divided doses to avoid the physiologic abnormalities associated with single doses.

### Regulatory and administrative barriers to appropriate dosing

There were studies as early as 1985 documenting accelerated methadone metabolism in pregnancy (20) and improved outcomes using divided doses of methadone (21). These had little impact on actual dosing of most pregnant women who continued to be prescribed methadone as a single dose. Until recently, Federal regulations required an exception to provide methadone using a divided dose regimen. This was not often used because programs were discouraged from giving daily take home doses because of exaggerated fears of diversion. For decades, therefore, most pregnant women have been dosed without regard for their unique metabolic state and without awareness of adverse effects of incorrect dosing on the fetus. This continues to be a serious problem in the highly regulated methadone treatment system.

The fact that these early studies on the need and benefit of split dosing were largely ignored speaks of how effective and safe provision of methadone during pregnancy has been discouraged by Federal regulations posing barriers to divided dosing, and further undermined by the view of the mother as someone who cannot be trusted with take home doses. Programs still refuse to split-dose pregnant women because they do not trust the mother not to divert the medication. Mis-dosing mother and fetus is therefore justified as preventing hypothetical diversion. This is a myth based on the view of the mother as someone who does not care for the wellbeing of her baby and who would sell the methadone, which she knows is critical to keeping her baby out of withdrawal. These mothers have normal protective concerns about the safety of the baby and, therefore, are highly motivated to recover. However, conception often occurs during a period of use and dependence, and women can suffer temporary impairments in judgement and face significant barriers to accessing care. However, once in appropriate care, they are as motivated and able to have a healthy pregnancy as any other mother. They would not deliberately put their baby in withdrawal by diverting their methadone. Yet program biases about maternal “untrustworthiness” are still allowed to interfere with appropriate prescribing of methadone.

A study of the Subjective Opioid Withdrawal Scale (SOWS) augmented with two pregnancy-related items (uterine cramping and fetal hyperactivity) demonstrated that mothers are very aware of fetal hyperactivity when they themselves are experiencing withdrawal (10). When the pregnant women are inappropriately dosed, they feel compelled to use illicit opioids to treat their own and their baby's withdrawal. It reflects a serious failure of the methadone system when inappropriate dosing drives drug use rather than resolving it. It illustrates just one reason why mothers should have options for methadone treatment outside the clinic system, which often prioritizes administrative polices over proper medical care. NIDA director, Dr. Nora Volkow, has recently called for trained physician prescribing of methadone under a new regulatory system. Patients would then have options in choosing their care, options they do not have now. Addiction trained family medicine and obstetrical physicians would be an important starting point in this process of expanding access to methadone (22). A new SAMHSA ruling (April 28, 2022) has eliminated the need for special exceptions and all pregnant patients in the methadone system can now receive proper dosing solely at physician discretion. This will, hopefully, lead to changes at the level of State regulatory agencies and especially programs themselves whose risk management practices often prevent take home doses as a “program risk” that outweighs the medical needs of the mother and baby.

### Laboratory advances and dosing decisions

A newly available laboratory measure of metabolic activity is the serum methadone/metabolite ratio (MMR). This simple numeric ratio measures the speed of metabolism of the parent drug, methadone, to its inactive metabolite (23). Two studies have found a mean MMR of approximately 12 in a random population of methadone-maintained patients (24, 25). “Normal” metabolizing patients would have an MMR clustered around a mean of roughly 12 molecules of methadone to one of metabolite, within a “normal MMR range” of 8–16. Rapid metabolizers will have lower MMRs, and slow metabolizers will have higher ones. Eap et al. found a seventeen-fold variability in human metabolism of methadone (26). A study of 1,700 patients found an MMR range from 2 to 26, corroborating this wide range of methadone metabolism (23).

A study of the MMR in pregnancy demonstrated accelerated metabolism starting in the first trimester (mean MMR of 7. 2) which decreased to 5.9 in the second trimester, and then further decreased to 5.1 in the third trimester. The MMR then rose to 7.2 in the first two weeks post-partum, documenting a rapid reversal of metabolic induction (8). When the MMR is performed serially during the pregnancy, both physician and mother can monitor the changes in her metabolic rate and the effect on serum levels and on fetal exposure. Mothers are always concerned when high doses are needed. Therefore, laboratory data are important to discuss as part of physician counseling.

Dose increases are done to manage breakthrough withdrawal within the limits of the therapeutic serum range of 150–600 ng/ml, and the dose regimen is increased from an initial twice daily dosing on induction, to doses divided 3–4 times a day, roughly proportionate to the speed of metabolism (i.e., the lower the MMR the more frequent the dosing regimen). Patients with low MMRs (in the 3–6 range), especially in the third trimester, usually require 4–6 doses for optimal stability. Further, increasing the frequency of dosing may minimize the need for increased doses by providing the medicine more effectively. Finally, it is unknown what effect high level fentanyl dependence has on the efficacy of this serum level range, which was an effective guide for heroin and opioid pill dependence. The current therapeutic range needs further study and perhaps modification.

### Compassionate, supportive care of the mother

The stress on a pregnant woman who is opioid dependent can be quite severe, encompassing anxiety and guilt about effects of their drug use on the baby, confusion about the medication and possible adverse effects on the baby (especially NAS), family pressures to stop the medication, and the need to conceal use of methadone treatment for fear of condemnation by family and friends. It is a very lonely experience that is best overcome by a close on-going supportive relationship using a pregnancy team approach with a pregnancy-trained counselor, a nurse practitioner or physician assistant who usually manages acute care, and regular supportive meetings with the prescribing physician. This approach of comprehensive care of the pregnant woman with SUD was described forty years ago (27). Frequent contacts with the physician are needed, not only to manage changing dosing needs, but also to discuss issues such as hospital care, NAS risks, breastfeeding, dose reductions post-partum, potential interactions with child protective services, and concurrent mental health issues which are ideally but rarely managed within the methadone program.

High anxiety and stress states are associated with adverse outcomes independent of substance use (19). Yet maternal stress is rarely considered as a factor in poor outcomes to be addressed as a component of good methadone care. Methadone programs promote “non-medical counseling” as what they offer to help recovery. They do not mention close physician/patient contact because that is not the usual methadone model. While it is not possible to quantitate the effect of these factors in fetal outcomes, it is reasonable to expect that supportive physician interventions can mitigate stress. Knowing that the physician prescribing methadone is available and willing to confer with obstetricians and neonatologists on the patient's behalf can significantly reduce adverse effects of stress on the birthing process and improve outcomes. Such “medical counseling” would be ideally done by trained obstetricians or family medicine physicians, if they were allowed to use methadone, as they are allowed to use buprenorphine.

There is an urgent need to change Federal regulations that limit access to methadone to clinics that meet only 10%–15% of the national need and impose burdens of excessive attendance that interferes with job, school, childcare, and family obligations, in addition to increasing risks of exposure to viral infections in overcrowded clinics. Proposals for physician prescribing, pharmacy dispensing of methadone and ending the clinic monopoly on care are currently under consideration and are urgently needed to address the opioid overdose crisis (28). This urgency is illustrated by situations where pregnant patients are prescribed methadone as a single dose by programs unaware of current science and the availability of exception process. Pregnant patients in the clinic system can be significantly over-sedated on single doses and yet are denied appropriate dosing, even when it threatens their ability to care for their children.

### Maternal infant separation policies and criminalization of NAS

Time has also clarified the actual factor responsible for the severity of NAS in methadone exposed neonates described repeatedly in the media and in medical literature. Separation of mother and neonate and overuse of NICUs has been shown to contribute to the expression of NAS and prolonged hospitalizations. Once neonatologists and obstetricians stopped separating mothers and babies, the rates of NAS treatment and length of stay in hospital fell dramatically (29–31). Numerous reports have also clarified the other issues that affect the expression of neonatal abstinence, including, breastfeeding (32), genetics (33), pharmacokinetics (8), smoking (34), gestational age (2) and others. The signs of NAS can mimic those of other serious diseases such as, sepsis, cerebral hemorrhage, hypoglycemia and hypocalcemia; these disorders will need to be ruled out. The seminal paper on NAS by Finnegan et al. did not mandate NICU care and used the term “comprehensive care” for the needs of the maternal/fetal dyad (27). This term was only “rediscovered” recently as part of the recognition of the critical importance of compassionate care and the importance of the mother in the amelioration of NAS (5).

The idea that NAS was so severe that NICU care was virtually mandatory evolved during the era of drug war polemics. Since drug users were labelled as criminals, and pregnant drug users were labelled “child abusers”, then the NICU was a way of protecting the newborn from an inadequate mother. It was as much a social punishment as a medical intervention. However, in the early years when opioid withdrawal was a new diagnosis with a high mortality, medical protection of the baby was important and nurses with the best training were in NICU's.

However, separation of the mother from her newborn involved ignoring what is known scientifically about the critical importance of early maternal/infant skin-to-skin contact, which promotes reciprocal hormonal interactions critical to attachment and to managing the physiology of NAS. NICU care made breastfeeding very difficult, yet breastfeeding was shown to mitigate NAS (32). The two neurohormones that are especially critical are endorphin and oxytocin, which are stimulated by nursing and skin-to-skin contact. To deprive the newborn of this critical process by placing the baby in a NICU reflects how the social anti-drug milieu affected medical judgement. And this bias was what created the nationwide epidemic of “severe NAS” which made headlines in every newspaper across the country, and for which methadone was widely blamed.

The State of Tennessee went so far as to criminalize having a baby who had NAS, resulting in women being coerced into attempting rapid methadone withdrawal. An outcome study of this process was published purporting to find that an ultra-rapid withdrawal was “not harmful” because there were no “apparent complications” beyond two fetal deaths during jail withdrawals (35). Minimal monitoring of maternal and fetal stress was done and there was no long-term follow up of the mother or baby. This attempt to put pregnant women through a potentially life-threatening opioid withdrawal without intensive maternal/fetal monitoring has been called “stressing the fetal brain” (36). Pregnant incarcerated women are often forced through abrupt opioid withdrawal, some of whom predictably experience miscarriages. These fetal deaths are a direct result of law enforcement bias against methadone and their refusal to allow women to access it. They see their role as punishing mothers even though it is traumatic to the fetal brain, can cause epigenetic modifications and long-term developmental problems, and can result in fetal death (17, 18).

### Ending the inadequate mother model

The first change in the separation model of NAS management came from England where Saiki et al. reported on a hospital policy change that mandated the maternal/neonatal dyad should be kept together on the regular maternity unit (29). This resulted in an 11% rate of treatment for NAS and a reduction of duration of hospital care from 12.7 to 7.3 days. In the US, Holmes et al. introduced the term “rooming-in” for the new model of care relying primarily on the mother to mitigate symptoms and found similar reductions in number of neonates treated, length of stay, and cumulative morphine doses in both methadone and buprenorphine exposed neonates (30). These authors concluded that “the environment of care is likely more important than the medication used for treatment”. Grossman et al. furthered this rooming-in approach with a new NAS assessment tool termed “Eat, Sleep, and Console” (ESC), simplifying target symptoms for medication usage (appropriate eating, sleeping, and control of distress) (37). Over a 5-year period this model reduced the use of any morphine from 98% to 12% and reduced the length of stay from 22.5 to 5.9 days. Eighty percent of patients in this cohort were on methadone. In a recent cluster trial, the ESC approach was associated with shortened length of stay and duration of treatment (38). Results are indeed in support of the importance of the environment in the management of the mother-infant dyad but the study awaits long-term follow-up (39).

### Summary

Aspects of methadone use in pregnancy that reduce risks for NAS symptoms include the systematic use of methadone serum levels, especially trough levels and MMRs, to guide dosing during the metabolically dynamic perinatal period. This includes routine use of split-dosing to minimize adverse fluctuations of serum levels associated with withdrawal and fetal side effects. Dosing decisions should be discussed with the mother in the context of regular physician counseling, education, and stress management. Post-partum hospital care relying primarily on normal mother/infant comforting and nurturing interactions for control of withdrawal symptoms will further minimize NAS symptoms.

Studies to date have compared single daily maternal dosing with methadone and daily dosing of buprenorphine regarding the amount of morphine needed to treat NAS, duration of treatment for NAS and length of stay. Former studies demonstrated a significant difference between methadone and buprenorphine regarding the three outcomes listed above. The question is did these differences occur because of a real difference between the two medications or was the single dosing of methadone the main issue. Future studies need to be directed to comparing the effects of split versus single dosing of methadone and buprenorphine regarding newborn outcomes including the same parameters as evaluated in the initial studies (40). Studies should describe not only the actual methadone treatment practices including dosing practices and serum levels, but also the availability and nature of physician support, and hospital practices for NAS management so that outcomes are not skewed by the adverse effects of either unphysiological dosing, lack of physician support, or maternal/infant separation. This should establish more accurately the real risks of NAS under these optimal methadone dosing conditions.

Furthermore, methadone, a full agonist, is pharmacologically, a more appropriate medication for use in medication induction in pregnancy during the current epidemic of fentanyl-dependent pregnant women. Methadone avoids the risks to the fetus of harm from precipitated buprenorphine withdrawal and the need for polypharmacy to manage such withdrawal (41–43). This emphasizes the critical importance of changing Federal regulations that prevent physicians, especially obstetricians and family medicine doctors trained in addiction medicine, from using a safer and more effective medication to manage severe dependence.
